# Laws of Genome Nucleotide Composition

**DOI:** 10.1093/gpbjnl/qzae061

**Published:** 2024-08-30

**Authors:** Zhang Zhang

**Affiliations:** National Genomics Data Center, China National Center for Bioinformation, Beijing 100101, China; Beijing Institute of Genomics, Chinese Academy of Sciences, Beijing 100101, China; University of Chinese Academy of Sciences, Beijing 100049, China

Genome nucleotide composition, one of the most important sequence characteristics at the genome-wide level, is usually expressed in terms of the proportions of four bases in DNA molecule as well as their combinations. It has been studied for decades that genomes of different species are highly variable in their nucleotide composition [1,2], as demonstrated that guanine-plus-cytosine (GC) content varies widely with a broader range from ∼ 20% to ∼ 80% [3]. A body of empirical evidence has further accumulated that heterogeneity of genome-wide nucleotide composition in different species associates closely with a variety of intrinsic and extrinsic factors, such as genome size [4], phylogeny [5], growth temperature [6], environment [7], origin of replication [8], bacterial land colonization [9], codon/amino acid usage [10], and natural selection [11]. Contrastingly, very few theoretical efforts have been devoted to exploring whether there is any law underlying such variable genome nucleotide composition across different species. Theoretically, such law(s) would be desirable to be used as a fundamental framework for better understanding genome composition dynamics, molecular evolution, genome organization, and synthetic biology. Built upon previous findings, here we propose three laws of genome nucleotide composition in a mathematical manner and demonstrate their effectiveness to formulate diverse genome nucleotide compositions in a large collection of complete genome sequences across three domains of life.

## First law: the law of base pairing

**Figure 1:**
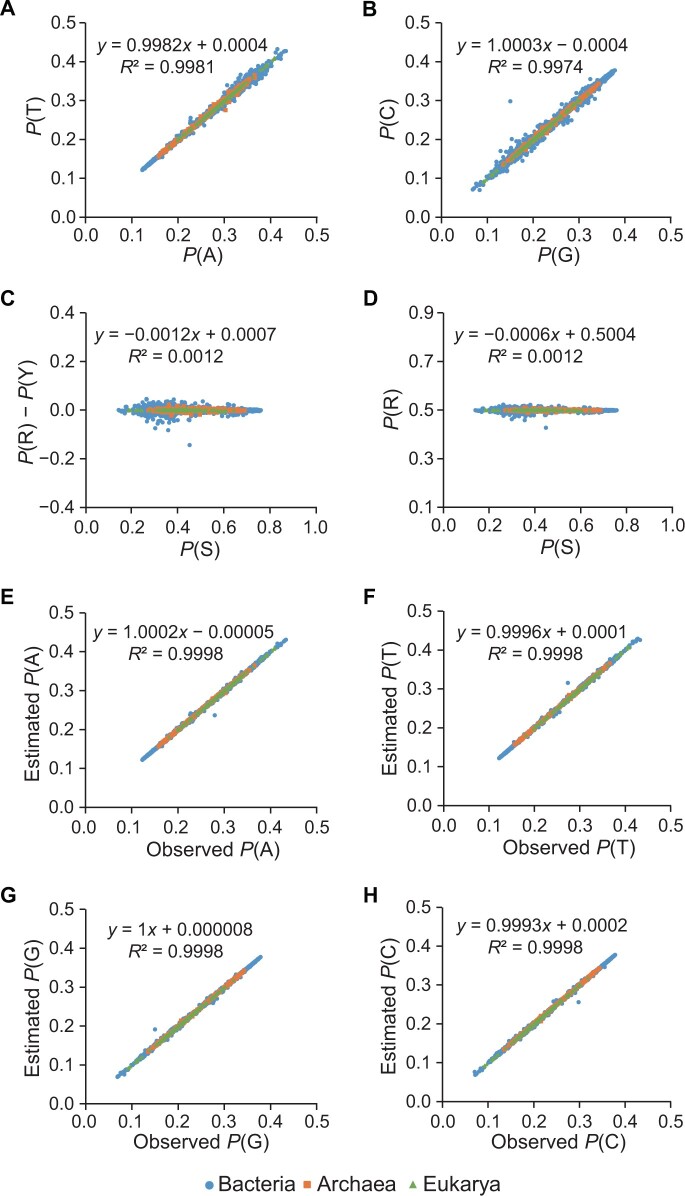
Genome-wide proportion of nucleotide compositions across three domains of life **A**. Proportion of A *vs*. proportion of T. **B**. Proportion of G *vs*. proportion of C. **C**. Proportion of S *vs*. difference between proportion of R and proportion of Y. **D**. Proportion of S *vs*. proportion of R. **E**. Observed *vs*. estimated proportions for A. **F**. Observed *vs*. estimated proportions for T. **G**. Observed *vs*. estimated proportions for G. **H**. Observed *vs*. estimated proportions for C. A total of 17,873 complete genomes were obtained from NCBI RefSeq, including 313 in Archaea, 17,289 in Bacteria, and 271 in Eukarya. Observed proportions of the four bases were derived from these genome sequences and estimated proportions of the four bases were quantified according to Equations 5–8. In all panels, each point represents a specific genome. An obvious outlier in panels B to G is *Candidatus* Chazhemtobacterium aquaticus Ch65, with genome size at 801,504 bp and *P*(A) = 27.87%, *P*(T) = 27.33%, *P*(G) = 14.97%, and *P*(C) = 29.83%. A full list of their genome-wide nucleotide compositions is tabulated into Table S1. NCBI, National Center of Biotechnology Information; RefSeq, Reference Sequence Database; A, adenine; T, thymine; G, guanine; C, cytosine; S, guanine-plus-cytosine content; R, purine content; Y, pyrimidine content; *P*, proportion.

The first law is Chargaff’s rules [12] that adenine (A) pairs with thymine (T) and guanine (G) pairs with cytosine (C), leading to *P*(A) = *P*(T) and *P*(G) = *P*(C), where *P* is the proportion (probability) of any base as well as their combination. Such base pairing symmetry in any double-stranded genome and each single strand corresponds to the Chargaff’s first parity rule and second parity rule, respectively. With biological, chemical, and physical significances, the rules played a crucial role in the discovery of the double helix structure of DNA in 1953 and laid profound foundations in advancing molecular biology and genomics. Despite the debate on the Chargaff’s second parity rule, the first law holds valid as testified by nearly perfect linear regression in a wide diversity of genomes across the three domains of life (**Figure 1**A and B). In what follows, *P* is calculated based on single strands of genomes.

(1) P(A)=P(T)

(2) P(G)=P(C)

## Second law: the law of base equality

The second law states the base equality between purines (A and G) and pyrimidines (T and C) derived from the first law or the Chargaff’s second rule. As a consequence of the base pairing nature of DNA double helix, a 1:1 stoichiometric ratio of purines (R) and pyrimidines (Y) can be deduced. In other words, the proportion of R approximates the proportion of Y, namely, *P*(R) = *P*(Y). Conforming with the second law as well as previous findings [13], *P*(R) is observed to approximate *P*(Y) and fluctuate around 50% in diverse genomes (Figure 1C and D).

(3) P(R)=P(Y)=50%

(4) P(S)+P(W)=100%

Meanwhile, since independence of two variables means that the occurrence of one variable does not affect the probability of the other, GC content (S) or AT content (W), varying from ∼ 20% to ∼ 80%, is believed to be statistically independent from R or Y, as indicated by linear regression slope very close to the optimum value of zero and intercept near 0.5 (Figure 1D).

## Third law: the law of base composition

The third law expresses the principle of base composition. Suppose that the universal set of the four bases is Ω = {A, T, G, C} and X and Y are two subsets of Ω, there are three common set operations: (1) union of X and Y, *viz*., X∪Y, is the set of all elements that are members of X or Y or both; (2) intersection of X and Y, *viz*., X∩Y, is the set of all elements that are members of both X and Y; (3) complement of X relative to Ω, *viz*., X^c^, is the set of all members that are not members of X. Thus, GC and purine contents can be denoted as S = G∪C and R = A∪G, respectively. Likewise, W = A∪T = S^c^ and Y = T∪C = R^c^. Because S and R form a statistically independent pair as mentioned above, *P*(S∩R) can be quantitatively expressed as *P*(S∩R) = *P*(S) × *P*(R), which is also applicable to the other three statistically independent pairs: S^c^ and R, S and R^c^, and S^c^ and R^c^ (for details see our previous study [14]). As a result, the proportion of each base can be quantitatively formulated as:

(5) $$P\left(A\right)=P\left(S^{c}\cap R\right)=P\left(S^{c}\right)P\left(R\right)$$

(6) $$P\left(T\right)=P\left(S^{c}\cap R^{c}\right)=P\left(S^{c}\right)P\left(R^{c}\right)$$

(7) P(G)=P(S∩R)=P(S)P(R),

(8) $$P\left(C\right)=P\left(S\cap R^{c}\right)=P\left(S\right)P\left(R^{c}\right),$$

where *P*(S^c^) = *P*(W) = 1 − *P*(S) and *P*(R^c^) = *P*(Y) = 1 − *P*(R) according to Equations 3 and 4. Strikingly, if *P*(R) = 0.5, then *P*(A) = *P*(T) = *P*(S^c^)/2 and *P*(G) = *P*(C) = *P*(S)/2, which is a special case equivalent to the first law or Chargaff’s rules. Based on our empirical datasets, the third law is effective to quantify the base proportions very close to the observed ones, as signified by squared correlation coefficients very close to the optimum value of 1.0, linear regression slopes near the optimum value of 1.0, and intercepts approaching the optimum value of zero (Figure 1E–H). Taking *Saccharomyces cerevisiae* S288C (assembly accession: GCF_000146045.2) as an example, *P*(S) = 0.3815 and *P*(R) = 0.5004. Thus, the estimated proportions are *P*(A) = *P*(S^c^) × *P*(R) = (1 − 0.3815) × 0.5004 = 0.3095, *P*(T) = *P*(S^c^) × *P*(R^c^) = (1 − 0.3815) × (1 − 0.5004) = 0.3090, *P*(G) = *P*(S) × *P*(R) = 0.3815 × 0.5004 = 0.1909, and *P*(C) = *P*(S) × *P*(R^c^) = 0.3815× (1 − 0.5004) = 0.1906, which are very close to the observed ones, 0.3098, 0.3087, 0.1906, and 0.1909, respectively.

## Concluding thoughts

**Figure 2:**
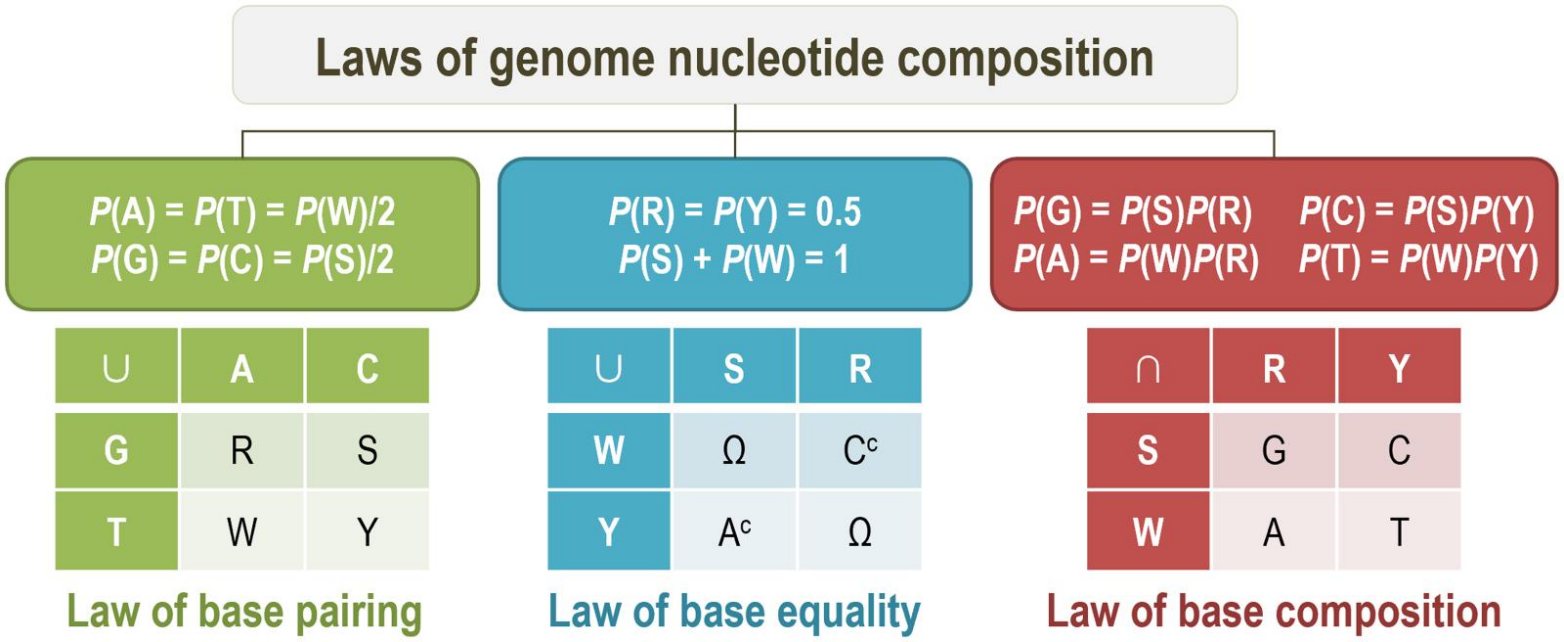
Schematic representation of laws of genome nucleotide composition The laws are illustrated by equations and operations of intersection (∩), union (∪), and complement (^c^) on four bases — A, T, G, and C, where *P* is the proportion (probability) of any base as well as their combination. The first law of base pairing states the complementary nature of DNA that leads to *P*(A) = *P*(T) = *P*(W)/2 and *P*(G) = *P*(C) = *P*(S)/2, where W = A∪T, S = G∪C, R = A∪G, and Y = T∪C. The second law of base equality reveals the quantitative relationships of *P*(R) = *P*(Y) = 50% and *P*(S) + *P*(W) = 100%, where R∪Y or S∪W is the universal set Ω = {A, T, G, C}, R∪W = {A, T, G} = C^c^, and Y∪S = {T, G, C} = A^c^. The third law deduces the mathematical principle of each base composition, given the independence relationship between S and R, *viz*., *P*(G) = *P*(S∩R) = *P*(S) × *P*(R), *P*(A) = *P*(W∩R) = *P*(W) × *P*(R), *P*(C) = *P*(S∩Y) = *P*(S) × *P*(Y), and *P*(T) = *P*(W∩Y) = *P*(W) × *P*(Y), where W = S^c^ and Y = R^c^. W, adenine-plus-thymine content.

To sum up, the three laws provide a unifying theoretical framework of genome nucleotide composition (**Figure 2**). The first law of base pairing uncovers the complementary nature of DNA that is essential for its structure, stability, and function; the second law of base equality reveals the equality relationship between purines and pyrimidines as well as the independence relationship between GC content and purine content; and the third law deduces the mathematical principle of each base composition. As validated in large-scale empirical genome sequences, the three laws are able to unravel the mystery of various genome-wide nucleotide compositions across diverse organisms. Meanwhile, it should be noted that the three laws are applicable to genome-level sequences, not genes or specific regions. Critically, as a theoretical framework, they can be used to design genomes with specified composition and detect organisms with unusual nucleotide composition that potentially experience complex evolutionary processes adapted to extreme environments [see one example in Figure 1B, as reported in the study by Kadnikov et al. [15] — *Candidatus* Chazhemtobacterium aquaticus Ch65, genome size at 801,504 bp, *P*(A) = 27.87%, *P*(T) = 27.33%, *P*(G) = 14.97%, and *P*(C) = 29.83%, with strong disparity between *P*(G) and *P*(C)].

As Leonardo da Vinci said: “He who loves practice without theory is like the sailor who boards ship without a rudder and compass and never knows where he may cast”. Nowadays (and in the foreseeable future), we are drowning in the deluge of multi-omics data, thirsting for fundamental theories and laws to explain or predict a range of biological phenomena that are derived from a number of empirical observations and experiments. From this perspective, the three laws potentially pave the way to a new era with quantitative insights for studying genome organization and evolution, driving synthetic genome engineering, and further advancing theoretical biology, with the ultimate goal for deciphering basic principles of life.

## CRediT author statement

**Zhang Zhang:** Conceptualization, Data analysis, Writing – original draft, Writing – review & editing, Project administration, Funding acquisition. The author has read and approved the final manuscript.

## Supplementary Material

qzae061_Supplementary_Data
