# Supplementary material for: Laws of Genome Nucleotide Composition
Source: Genomics Proteomics Bioinformatics. 2024 Aug 30;22(4):qzae061. doi: 10.1093/gpbjnl/qzae061 (PMC11514846; doi:10.1093/gpbjnl/qzae061)
Supplement: qzae061_Supplementary_Data [file qzae061_supplementary_data.zip › supplementary material captions.docx]

**Supplementary material**

**Table S1 Statistics of genome-wide nucleotide composition and related meta information of 17,873 complete genome sequences across three domains of life obtained from NCBI RefSeq**
